# Factors Affecting Sleep Quality Among Pregnant Women: A Propensity Score‐Matched Analysis

**DOI:** 10.1111/jog.70167

**Published:** 2025-12-11

**Authors:** Yinxia Zheng, Tian Chen, Reyila Yalihong, Moli Duan

**Affiliations:** ^1^ Urumqi Maternal and Child Health Hospital Urumqi China; ^2^ Xinjiang Clinical Research Center for Perinatal Diseases, Urumqi Maternal and Child Health Hospital Urumqi China; ^3^ School of Public Health Xinjiang Medical University Urumqi China; ^4^ Urumqi Youai Hospital Urumqi China

**Keywords:** pregnant women, propensity score matching, psychological stress, sleep quality

## Abstract

**Objective:**

To assess sleep quality in pregnant women and analyze its influencing factors based on lifestyle, stress levels, physical activity, and other relevant aspects.

**Methods:**

From August 2022 to July 2024 in Urumqi, Xinjiang, a random sampling method was used to assess sleep quality using the pittsburgh sleep quality index (PSQI). General demographic characteristics were collected from 3508 pregnant women. A 1:1 propensity score matching method was used to match participants with good and poor sleep quality, resulting in 1402 pregnant women in each group. The factors influencing sleep quality were analyzed using least absolute shrinkage and selection operator‐logistic regression.

**Results:**

Logistic regression analysis revealed that higher monthly income, no history of abortion, and lower exposure to passive smoking before pregnancy were protective factors for sleep quality, while high stress levels and conception via ART (assisted reproductive technology) were identified as risk factors.

**Conclusion:**

Stress and lifestyle habits significantly affect the sleep quality of pregnant women in Urumqi, Xinjiang. Reducing psychological stress through health education and encouraging the development of healthy behavioral habits potentially enhance sleep outcomes. However, due to the regional specificity of the sample, these findings should not be generalized to pregnant women in other regions with different ethnic, cultural, or environmental characteristics.

## 1 Introduction

Pregnancy induces several physiological changes in the body, including hormonal, anatomical, and mechanical adaptations. These changes affect various physiological functions, particularly sleep [1]. Studies show that many women experience changes in sleep timing, patterns, and quality during pregnancy. Additionally, these changes increase the risk of sleep disorders, including insomnia, obstructive sleep apnea, and restless leg syndrome, conditions that are often underrecognized by pregnant women [2].

Poor sleep quality, negatively affecting both fetal development and pregnancy outcomes [3], is more prevalent among women at risk for psychiatric disorders [4]. A recent review reports that 38.2% of pregnant women experience insomnia, 15% obstructive sleep apnea, and 20% restless leg syndrome. Sleep deprivation during pregnancy is associated with higher risks of preterm birth, cesarean delivery, high blood pressure, gestational diabetes, and prolonged labor [5]. Sleep disorders during pregnancy, associated with increased inflammation and gestational diabetes, along with poor sleep associated with preeclampsia, are recognized risk factors for the recurrence of mood disorders in women [6]. These mood disturbances further disrupt sleep patterns. Treating insomnia during the third trimester may alleviate symptoms of postpartum depression [7]. Effective management of these conditions could potentially reverse their adverse consequences.

A review of both domestic and international studies indicates that several factors influence sleep quality during pregnancy. These factors include age, marital status, occupation, gestational age, history of abortion, pregnancy‐related complications, number of births, smoking status, alcohol consumption, stress, anxiety, and depression [3]. Stress represents the nonspecific response of the body to external or internal demands, while a stressor refers to the stimulus that elicits this response. Stress is an inevitable aspect of modern life. World Health Organization (WHO) data indicate that 90% of the global population experiences stress. While moderate stress enhances motivation, prolonged or excessive stress may lead to negative emotions, impaired immune function, and disturbances in the nervous system [8]. To cope with these adverse effects, individuals often postpone self‐care or delay time for relaxation. International studies show that, after experiencing stressful events during the day, individuals often seek personal time at night, leading to the postponement of their bedtime to extend leisure activities and delay sleep onset [9]. Additionally, stress increases neural activity, inhibiting the transition from nervous system activation to relaxation and thereby making it difficult to fall asleep. Consequently, stress represents a significant factor influencing sleep.

Evidence from both domestic and international studies suggests that, whereas several studies have explored factors influencing sleep quality during pregnancy, several confounding factors affect sleep. This is particularly evident in Xinjiang, a multi‐ethnic region characterized by diverse cultural practices and lifestyles. Dietary habits, such as a preference for high‐fat meats and consumption of brick tea containing caffeine, may contribute to physical discomfort and disrupt sleep. Significant day–night temperature fluctuations and high levels of nighttime activity easily disrupt the circadian rhythms of pregnant women. Additionally, the distribution of medical resources in the region is uneven, limiting access to prenatal care for women in remote areas. Language barriers further inhibit effective communication with healthcare providers. These differences in ethnicity, culture, lifestyle, and healthcare infrastructure contribute to the unique sleep challenges experienced by pregnant women in Xinjiang.

A review of both domestic and international studies indicates that several factors influence sleep quality during pregnancy, including stress, adverse pregnancy history (e.g., abortion), lifestyle behaviors (e.g., passive smoking), and ART (Assisted Reproductive Technology) [3]. However, the unique ethnic, cultural, and environmental context of Xinjiang (e.g., dietary preferences for high‐fat meats, significant day‐night temperature fluctuations, and uneven medical resource distribution) may modify the association between these factors and pregnant women's sleep quality, which remains understudied.

Based on prior evidence and the regional characteristics of Xinjiang, we hypothesized that: (1) High psychological stress and conception via ART would be independent risk factors for poor sleep quality in pregnant women in Urumqi; (2) No history of abortion, lower exposure to passive smoking (before and during pregnancy), and higher monthly income would serve as protective factors for sleep quality in this population.

To test these hypotheses, we conducted a cross‐sectional study with propensity score matching to control for confounding variables (e.g., age, education, occupation), and used LASSO‐logistic regression to identify key influencing factors. The findings aim to provide region‐specific evidence for improving maternal sleep quality and reducing adverse maternal–infant outcomes in Xinjiang.

## 2 Materials and Methods

### 2.1 Study Population

An online questionnaire survey was conducted among pregnant women receiving medical care at Urumqi Maternal and Child Health Hospital from January 1, 2022, to June 3, 2024, including participants from multiple ethnic groups, such as Han, Hui, and Uyghur. On‐site staff were available to assist participants with low literacy or limited education. The inclusion criteria were as follows: (1) age ≥ 18 years; (2) regular participation in prenatal check‐ups; (3) intact cognitive function, with the ability to read, understand, and respond to survey questions; and (4) willingness to participate in the study. The exclusion criteria were as follows: (1) critically ill patients unable to complete the questionnaire; and (2) individuals with mental illness or cognitive impairment preventing effective participation. Overall, 3600 questionnaires were distributed, of which 3508 were valid, resulting in an effective response rate of 97.44%. The study was conducted following the Declaration of Helsinki. All participants provided written informed consent, were informed of their right to withdraw at any time, and were assured that their data would be strictly safeguarded by designated personnel. Ethical approval was received from the Ethics Committee of Urumqi Maternal and Child Health Hospital (approval number: XJFYLL2021032).

### 2.2 Questionnaire Survey

A self‐administered questionnaire was used to collect the following information: (1) general sociodemographic characteristics, including age, education level, occupation, economic status, and residence; (2) pregnancy‐related characteristics, such as number of pregnancies, live births, abortions, and mode of delivery; and lifestyle behaviors, including passive smoking status, physical activity, diet, stress; and (3) sleep quality assessed using the Pittsburgh Sleep Quality Index (PSQI) [10], developed by the University of Pittsburgh, Pennsylvania, in 1989. The PSQI is widely used to evaluate sleep quality in diverse populations, including both individuals with sleep disorders and healthy adults. In this study, the validated Chinese version of the PSQI was used, which has been psychometrically tested and widely applied in Chinese populations. The scale is a self‐administered questionnaire, comprising 18 items, each rated on a 0–3 scale. These items are grouped into seven components: (C1) sleep quality (subjective sleep quality score), (C2) sleep latency (time to fall asleep), (C3) sleep duration, (C4) sleep efficiency (proportion of time spent asleep while in bed), (C5) sleep disturbances (frequency of challenges associated with sleep), (C6) use of hypnotic drugs (frequency of use), and (C7) daytime dysfunction. The PSQI score (0–21 points) is the sum of the seven components, with higher scores indicating poorer sleep quality. A PSQI score > 5 is considered to indicate poor sleep quality [11].

#### 2.2.1 Measurement of Life Stress

Life stress was evaluated using a single self‐reported question: “How would you rate your current level of life stress?” Responses were recorded on a two‐point categorical scale with the following options: “Low” (indicating low perceived life stress) and “High” (indicating high perceived life stress). This measurement approach was selected primarily to reduce the response burden on pregnant participants, ensuring high completion rates and data quality (effective response rate: 97.44%) in a cross‐sectional survey setting. Additionally, it aligns with methods used in comparable studies on pregnancy and sleep quality, facilitating preliminary cross‐study comparisons.

The single binary item used to assess stress has inherent limitations: it cannot capture the gradient of stress intensity (e.g., mild, moderate, severe) or distinguish between stress types (e.g., pregnancy‐specific stress related to fetal health vs. general life stress). Thus, it may underestimate or overestimate the actual stress exposure of participants, limiting the nuance of stress‐related analyses.

### 2.3 Statistical Analysis

Data analyzed using IBM SPSS Statistics for Windows, Version 26.0 (IBM Corp., Armonk, NY, USA). The chi‐square test was used to compare differences between the two groups in sociodemographic characteristics, pregnancy‐related factors, and lifestyle behaviors. A propensity score matching model was used to perform 1:1 matching of 3508 pregnant women. A propensity score matching (PSM) model was used to perform 1:1 matching of 3508 pregnant women. PSM variable selection criteria: Variables were chosen based on prior literature and clinical relevance—age, household registration, education level, marital status, occupation, and per capita annual income were selected as matching covariates, as these factors have been consistently reported to confound the association between socioeconomic/demographic characteristics and pregnancy sleep quality.

After matching, these five variables were balanced across both groups. Least absolute shrinkage and selection operator (LASSO)–logistic multivariate regression was used to identify factors influencing sleep quality in pregnant women, with a significance threshold of *p* < 0.05.

### 2.4 Definitions of Key Terms

Mental labor refers to work primarily involving cognitive activities, such as reasoning, analysis, problem‐solving, creativity, information processing, or knowledge production, rather than physical or manual tasks. This type of work typically depends on specialized knowledge, intellectual skills, or mental faculties (e.g., critical thinking, memory, or abstract reasoning) to generate, process, or apply information, ideas, or solutions. Examples include professionals in fields such as education, research, healthcare (e.g., physicians, psychologists), law, engineering, writing, and management. The International Standard Classification of Occupations (ISCO), formulated by the International Labour Organization (ILO), classifies occupations as professionals, technicians, and managers primarily involved in mental work.

ART encompasses several medical procedures and techniques designed to assist individuals or couples experiencing infertility or subfertility in achieving pregnancy. These interventions may involve manipulating gametes (sperm and eggs) or embryos outside the body (in vitro) or facilitating fertilization and implantation within the body (in vivo) to address barriers to conception, including ovulatory disorders, tubal factor infertility, male factor infertility, or unexplained infertility. Common examples include in vitro fertilization (IVF), intracytoplasmic sperm injection (ICSI), gamete intrafallopian transfer (GIFT), and embryo cryopreservation, among others, following the International Committee for Monitoring Assisted Reproductive Technology (ICMART) and WHO Revised Glossary of ART Terminology.

## 3 Results

### 3.1 Demographic Matching Between the Two Initial Groups

Among the 3508 pregnant women included in the study (Data S1), 887 (25.29%) had poor sleep quality before propensity score matching. Most participants were aged 26–35 years, and the proportion of urban household registration was significantly higher than that of rural. Regarding occupation, the number of mental workers was significantly higher than that of manual workers. Additionally, significant differences were observed in income levels across different sleep conditions. After propensity score matching, 1402 pregnant women were successfully matched, with no significant differences in demographic characteristics observed between the two groups, indicating that the matched data are suitable for subsequent analyses. Table 1 provides detailed information.

**TABLE 1:** Comparison of sleep quality among pregnant women before and after propensity score matching.

Variable	Before matching (*n* = 3508)				After matching (*n* = 2804)			
	Good sleep quality *n* (%)	Poor sleep quality *n* (%)	*χ* ^2^/fisher	*p*	Good sleep quality *n* (%)	Poor sleep *n* quality (%)	*χ* ^2^/fisher	*p*
Age (years)			6.38	0.17			6.577	0.12
≤ 25	171 (6.52)	44 (4.96)			120 (4.27)	28 (1.00)		
26–30	845 (32.24)	263 (29.65)			666 (23.75)	197 (7.03)		
31–35	771 (29.42)	287 (32.36)			644 (22.68)	233 (8.31)		
36–40	250 (9.54)	91 (10.26)			204 (7.28)	74 (2.64)		
≥ 41	584 (22.28)	202 (22.77)			477 (17.01)	161 (5.74)		
Educational level			5.30	0.15			2.50	0.48
Junior high school or below	311 (11.90)	82 (9.20)			220 (7.85)	60 (2.14)		
High school/technical secondary	350 (13.40)	116 (13.10)			270 (9.63)	84 (3.00)		
College/undergraduate	1835 (70.00)	640 (72.20)			1508 (53.78)	507 (18.08)		
Master's degree or above	125 (4.80)	49 (5.50)			113 (4.03)	42 (1.50)		
Marital status			3.26	0.20			1.26	0.53
Single	60 (2.30)	15 (1.70)			39 (1.39)	13 (0.46)		
Married	2555 (97.50)	867 (97.70)			2066 (73.68)	676 (24.11)		
Divorced	6 (0.20)	5 (0.60)			6 (0.21)	4 (0.14)		
Occupational type			8.3	< 0.01			4.92	0.026
Manual labor	167 (6.40)	82 (9.20)			152 (5.42)	68 (2.43)		
Mental labor	2454 (93.60)	805 (90.80)			1959 (69.86)	625 (22.29)		
Annual income (¥)			71.16	< 0.01			30.92	< 0.01
≤ 10 000	340 (13.00)	142 (16.00)			316 (11.27)	114 (4.07)		
10 001–30 000	759 (29.00)	333 (37.50)			722 (25.75)	296 (10.56)		
30 001–50 000	663 (25.30)	251 (28.30)			569 (20.29)	180 (6.42)		
≥ 50 001	859 (32.80)	161 (18.20)			504 (17.97)	103 (3.67)		

*Note:* Sleep quality was assessed by the Pittsburgh Sleep Quality Index (PSQI): PSQI score > 5 was defined as poor sleep quality, and PSQI score ≤ 5 as good sleep quality. Propensity score matching (PSM) was performed with a 1:1 ratio, and matching covariates included age, household registration, education level, marital status, occupation, and per capita annual income. Before matching, the total sample size was 3508; after matching, the total sample size was 2804 (1402 cases in each sleep quality group).

### 3.2 Pregnancy Characteristics and Lifestyle Factors of Pregnant Women After Matching

The number of pregnancies was mainly 1, while the majority had 0 and 1 previous birth or abortion, both accounting for 90.08%. Natural conception was the predominant method of pregnancy, followed by ovulation induction and ART, with ART accounting for only 1.14%, 3.67%, and 10.09% of pregnancies complicated by diabetes. Passive smoking occurred in 15.60% of women before pregnancy and 15.50% during pregnancy. Approximately 46.18% of participants reported engaging in regular exercise during pregnancy. Tables 2 and 3 provide detailed information.

**TABLE 2:** Characteristics associated with sleep quality among pregnant women after propensity score matching.

Variable	Good sleep quality, *n* (%)	Poor sleep quality, *n* (%)	*χ* ^2^/fisher	*p*
Number of pregnancies			28.81	< 0.001
1	1125 (40.12)	295 (10.52)		
2	545 (19.44)	211 (7.52)		
≥ 3	411 (14.66)	187 (6.67)		
Number of births			6.79	0.147
0	1240 (44.22)	370 (13.20)		
1	670 (23.89)	253 (9.02)		
≥ 2	201 (7.17)	70 (2.50)		
Number of abortions			38.01	< 0.001
0	1508 (53.78)	421 (15.01)		
1	413 (14.73)	184 (6.56)		
≥ 2	190 (6.78)	88 (3.14)		
Multiple births			15.40	< 0.001
Multiple	42 (1.50)	33 (1.18)		
Single	2069 (73.79)	660 (23.54)		
Mode of conception			22.506	< 0.001
Spontaneous conception	2061 (73.50)	651 (23.22)		
Ovulation induction	18 (0.64)	14 (0.50)		
ART	32 (0.14)	28 (1.00)		
Disease before pregnancy			15.433	0.004
Hypertension	14 (0.50)	14 (0.50)		
Diabetes	29 (1.03)	5 (0.18)		
Other	47 (1.68)	24 (0.86)		
No	2021 (72.08)	650 (23.18)		
Disease during pregnancy			7.533	0.057
Hypertension	67 (2.39)	36 (1.28)		
Diabetes	208 (7.42)	75 (2.67)		
Other	62 (2.21)	24 (0.86)		
No	1774 (63.27)	558 (19.90)		

*Note:* This table presents pregnancy‐related characteristics (e.g., number of pregnancies, mode of conception) of 2804 pregnant women after 1:1 propensity score matching (1402 cases in the good sleep quality group, 1402 cases in the poor sleep quality group). “ART” refers to Assisted Reproductive Technology. Sleep quality was defined by PSQI score (≤ 5 = good, > 5 = poor).

Abbreviation: LASSO, least absolute shrinkage and selection operator.

**TABLE 3:** Differences in lifestyle habits by sleep quality after propensity score matching.

Variable	Good sleep quality, *n* (%)	Poor sleep quality, *n* (%)	*χ* ^2^/fisher	*p*
Passive smoking before pregnancy			29.879	< 0.001
Everyday	283 (10.10)	153 (5.50)		
Occasionally	1828 (65.20)	540 (19.30)		
Passive smoking during pregnancy			40.920	< 0.001
Everyday	276 (9.80)	161 (5.70)		
Occasionally	1835 (65.40)	532 (19.10)		
Physical exercise			6.022	0.014
Yes	947 (33.77)	348 (12.41)		
No	1164 (41.51)	345 (12.30)		
Exercise frequency (per week)			4.36	0.360
< 1	41 (6.16)	49 (7.36)		
1–2	155 (23.27)	172 (25.83)		
3–4	68 (10.21)	77 (11.56)		
Everyday	48 (7.21)	56 (8.41)		
Exercise duration			11.52	0.010
< 30 min	165 (24.77)	151 (22.67)		
30–60 min	128 (19.22)	181 (27.18)		
≥ 60 min	19 (2.85)	22 (3.30)		
Taste preference			16.230	< 0.001
Saltish	138 (4.90)	68 (2.40)		
Normal	1688 (60.20)	505 (18.00)		
Light/low‐salt diet	285 (10.20)	120 (4.30)		
Diet type			3.407	0.182
Greasy	119 (4.20)	49 (1.70)		
Normal	1499 (53.50)	500 (17.80)		
Light	493 (17.60)	144 (5.10)		
Stress			400.303	< 0.001
Low	1284 (45.79)	118 (4.21)		
High	827 (29.49)	575 (20.51)		

Abbreviations: ART, assisted reproductive technology; B, regression coefficient; CI, confidence interval; SE, standard error.

### 3.3 Identification of Factors Influencing Sleep Quality in Pregnant Women Using the Least Absolute Shrinkage and Selection Operator Regression

**FIGURE 1:**
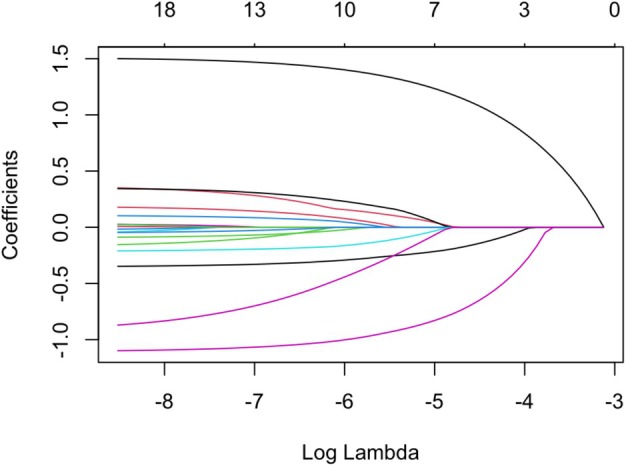
LASSO coefficient path plot. Each line (from top to bottom) represents: Stress, number of abortions, mode of conception, number of pregnancies, educational level, number of births, age, pregnancy complications, taste preference, dietary habits, physical activity, multiple pregnancies, marital status, monthly income, passive smoking before pregnancy, occupation type, passive smoking during pregnancy. Abbreviation: LASSO, least absolute shrinkage and selection operator.

Given the large number of variables in this study, LASSO regression was used to perform variable selection and reduce multicollinearity. Seventeen variables potentially affecting sleep quality in pregnant women, including demographic characteristics, pregnancy status, and lifestyle factors, were included in the LASSO regression model. Cross‐validation was used to determine an optimal penalty coefficient (*λ*) of 0.789. Finally, seven potential influencing factors were selected: monthly income, job type, number of abortions, pregnancy mode, passive smoking before pregnancy, passive smoking during pregnancy, and stress (Figure 1). Figure S1 showed the cross‐validation curve of LASSO regression analysis.

This receiver operating characteristic (ROC) curve evaluates the discriminative power of the binary logistic regression model, which included 7 variables screened by LASSO regression. The area under the ROC curve (AUC) was 0.715, indicating moderate ability to distinguish between pregnant women with good and poor sleep quality (PSQI ≤ 5 vs. PSQI > 5) (Figure S1).

### 3.4 Multivariate Analysis of Factors Influencing Sleep Quality in Pregnant Women

**FIGURE 2:**
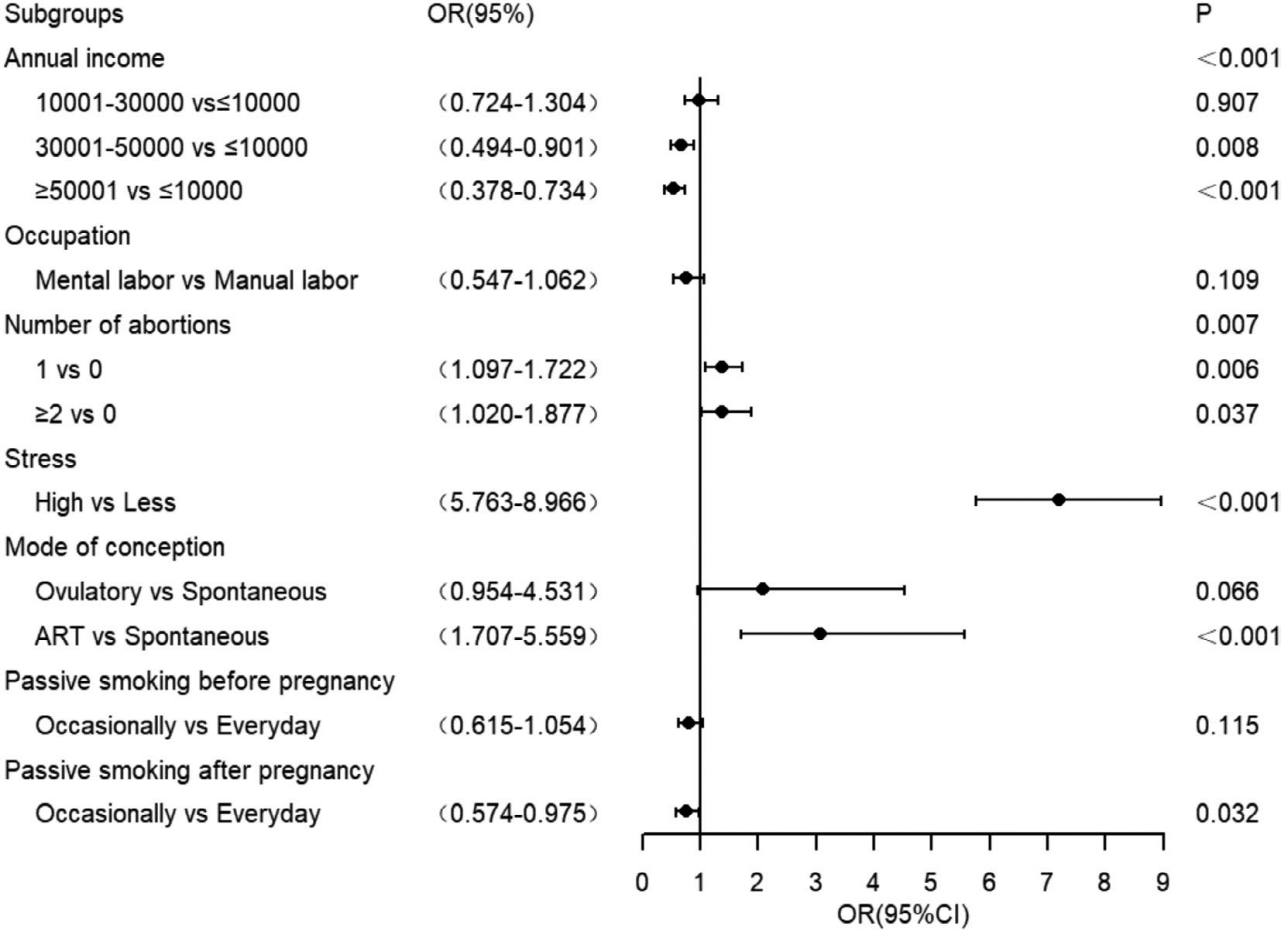
Forest plot showing factors associated with sleep quality in pregnant women. Abbreviations: ART, assisted reproductive technology; OR, odds ratio.

The seven factors identified using LASSO regression were included in a binary logistic regression model. The results showed that higher monthly income, no history of abortion, and lower exposure to passive smoking before pregnancy were protective factors for sleep, whereas high stress and ART were risk factors. Compared with pregnant women with a monthly income < 1000 yuan, those with a monthly income of 3001–5000 and > 5000 had better sleep quality (OR = 0.667, 95% CI: 0.494 ~ 0.901; OR = 0.527, 95% CI: 0.378–0.734, respectively). Having one or two abortions was identified as a risk factor for poor sleep quality (OR = 1.375, 95% CI: 1.097–1.722; OR = 1.383, 95% CI: 1.020–1.877). Compared with natural conception, pregnant women who conceived via ART exhibited lower sleep quality (OR = 3.081, 95% CI: 1.707–5.559). Lower exposure to passive smoking during pregnancy was associated with improved sleep quality (OR = 0.748, 95% CI: 0.574–0.975). High stress was a strong risk factor for poor sleep quality (OR = 7.188, 95% CI: 5.763–8.966). Table 4 and Figure 2 present detailed information.

**TABLE 4:** Multivariate logistic regression analysis of factors associated with sleep quality in pregnant women.

Variable	*B*	SE	Wald	*p*	Exp (*B*)	95% CI	
Annual income (≤ 10 000 [reference])			27.225	< 0.001			
10 001–30 000	−0.017	0.144	0.014	0.907	0.983	0.742	1.304
30 001–50 000	−0.404	0.153	6.949	0.008	0.667	0.494	0.901
≥ 50 001	−0.640	0.169	14.333	< 0.001	0.527	0.378	0.734
Occupation type (manual labor [reference])							
Mental labor	−0.271	0.169	2.573	0.109	0.762	0.547	1.062
Number of abortions (0 [reference])			10.041	0.007			
1	0.318	0.115	7.663	0.006	1.375	1.097	1.722
≥ 2	0.324	0.156	4.344	0.037	1.383	1.020	1.877
Stress (low [reference])							
High	1.972	0.113	306.01	< 0.001	7.188	5.763	8.966
Mode of conception (spontaneous [reference])							
Ovulation induction	0.732	0.397	3.391	0.066	2.079	0.954	4.531
ART	1.125	0.301	13.953	< 0.001	3.081	1.707	5.559
Passive smoking before pregnancy (everyday [reference])							
Occasionally	−0.216	0.137	2.478	0.115	0.805	0.615	1.054
Passive smoking after pregnancy (everyday [reference])							
Occasionally	−0.290	0.135	4.596	0.032	0.748	0.574	0.975

Discriminative ability: The area under the receiver operating characteristic curve (AUC) of the logistic regression model was 0.715 (Figure S2), indicating moderate discriminative power to distinguish between pregnant women with good and poor sleep quality.

Model fit: The Hosmer‐Lemeshow test was conducted to evaluate goodness‐of‐fit (*χ* ^2^ = 8.26, *p* = 0.40), which confirmed no significant difference between the observed and predicted values of the model, indicating an acceptable fit.

## 4 Discussion

Binary logistic regression analysis in this study revealed key factors affecting sleep quality among pregnant women in Urumqi, Xinjiang: high psychological stress and conception via assisted reproductive technology (ART) were identified as risk factors for poor sleep quality, whereas higher monthly income, no history of abortion, and lower pre‐pregnancy passive smoking exposure were protective factors that contributed to better sleep quality.

The overall incidence of poor sleep quality in this cohort was 25.29%, which is lower than that reported in other domestic and international studies (e.g., 34.14% in early pregnancy in a Chinese prospective cohort [12], 58.3% in early‐middle pregnancy [13]; a meta‐analysis shows 54.3%, 49.3%, and 69.6% prevalence in early, middle, and late gestation respectively [14]). Sleep is an essential physiological activity for human health, and pregnancy represents a unique period in a woman's life—physical and psychological changes during this stage increase the risk of fatigue, lethargy, and poor sleep, with prior evidence linking poor sleep quality to adverse outcomes like preterm birth and cesarean delivery [15, 16]. The associations identified in this study provide region‐specific insights into factors shaping sleep quality in Urumqi's pregnant population.

Higher income may alleviate financial stress related to childbearing and childrearing, thereby potentially improving sleep quality. In this study, pregnant women with a history of one or more miscarriages had a 1.3–1.4 times higher risk of sleep disorders than those without such a history; concern regarding another miscarriage may increase psychological pressure, leading to difficulty falling asleep—psychological interventions and family support can significantly alleviate these concerns [17]. The detrimental effects of secondhand smoke during pregnancy are well established: this study showed daily passive smoking exposure (affecting 15.5% of pregnant women in Xinjiang) was associated with sleep disorders, consistent with findings by Ma Feifan and Li Xingyi et al. [11, 18]. This may be attributed to nicotine, tar, and other tobacco byproducts inducing cellular proliferation, edema, epithelial thickening, and ciliary dysfunction [19].

Pregnant women who conceive via ART have a 3.08‐fold higher risk of sleep disorders than those with natural conception. Prior studies link ART to a higher risk of adverse perinatal outcomes, which may be associated with increased worry about fetal growth and development in ART‐conceived women—these factors together show an association with sleep disorders, but no causal relationship can be established [20]. Beyond ART, other psychological burdens, anxieties, and confounding factors may also negatively affect sleep.

Additionally, stress is a significant factor affecting sleep quality: 50% of pregnant women in this study reported higher stress levels (consistent with Ji Wenjia et al.'s finding that 71.47% of early pregnancy women experienced high stress [18]). Pregnant women face physiological changes, fear of childbirth/cesarean pain, concerns about fetal health, uncertainty about future life, and financial pressures—high stress may increase glucocorticoid secretion, hindering the nervous system from transitioning to a relaxed state and leading to poor sleep [21, 22, 23].

Notably, this study uses a cross‐sectional design, which cannot determine the direction of associations (e.g., high stress may link to poor sleep, or poor sleep may exacerbate stress, forming a bidirectional association); thus, no causal relationships between identified factors (stress, ART, abortion history) and sleep quality can be inferred, with conclusions limited to observed associations. The simple binary pressure measurement also prevents causal inference—future longitudinal studies should use multi‐item, valid stress scales (e.g., Perceived Stress Scale, PSS) to clarify this relationship's direction and extent [24].

Although key confounding factors were controlled via propensity score matching, residual imbalances in occupation type and annual income remained in the matched sample, potentially affecting internal validity and limiting generalizability across occupational/income groups. Furthermore, unmeasured confounders (history of mental illness, work environment [e.g., shift work], baseline sleep conditions before pregnancy) were not evaluated or included in the questionnaire/PSM model—these may affect sleep quality. Future studies could optimize the matching model to incorporate these factors and reduce bias. Additionally, the sample was limited to Urumqi, Xinjiang (a region with unique ethnic and cultural factors), so findings may not be generalizable to other populations.

Another key limitation is the limited generalizability of the findings. The study sample was restricted to pregnant women receiving prenatal care in Urumqi, Xinjiang—a region characterized by unique ethnic diversity (e.g., co‐residence of Han, Hui, and Uyghur populations), cultural practices (e.g., preference for high‐fat meats, consumption of caffeinated brick tea), and environmental conditions (e.g., significant day‐night temperature fluctuations). These region‐specific factors may interact with the identified sleep‐related factors (e.g., stress, passive smoking) in ways that differ from other regions in China or globally. For example, dietary habits or ethnic‐specific social support patterns in Urumqi may modify the association between stress and sleep quality, which may not be observed in non‐Xinjiang populations. Thus, the associations reported herein may not be applicable to pregnant women in other geographic or cultural contexts.

In summary, healthy behaviors, lifestyles, and dietary habits are closely associated with pregnant women's sleep quality. Obstetricians and gynecologists should proactively promote awareness of healthy interventions, provide balanced diet education, and guide the establishment of sustainable healthy habits to reduce adverse maternal and fetal outcomes.

## 5 Conclusion

Stress and lifestyle habits significantly impact sleep quality in Urumqi's pregnant women. Health education to reduce psychological stress and promote healthy behaviors may improve sleep outcomes, though findings are region‐specific and not generalizable to other ethnic, cultural, or environmental contexts.

For Xinjiang's maternal health policy, targeted actions include: integrating multilingual stress counseling (for Han, Hui, Uyghur) into prenatal care to address high stress (OR = 7.188); launching community anti‐passive smoking campaigns (focusing on rural areas, 15.6% pre‐pregnancy exposure) with low‐income subsidies (monthly income > 5000 yuan: OR = 0.527); and setting up specialized follow‐up for ART‐conceived women (OR = 3.081) via maternal health platforms. These align with Xinjiang's context, aiding sleep quality and reducing adverse maternal–infant outcomes.

## Author Contributions

**Yinxia Zheng:** writing – original draft, conceptualization, methodology. **Tian Chen:** data curation, formal analysis, software. **Reyila Yalihong:** investigation, validation. **Moli Duan:** writing – review and editing, conceptualization, project administration, formal analysis, supervision.

## Funding

The authors have nothing to report.

## Disclosure

The authors have nothing to report.

## Ethics Statement

Ethical approval was received from the Ethics Committee of Urumqi Maternal and Child Health Hospital (approval number: XJFYLL2021032).

## Consent

Informed consent was received from all participants.

## Conflicts of Interest

The authors declare no conflicts of interest.

## Supporting information


**Figure S1:** Cross‐validation curve of lasso regression analysis.
**Figure S2:** ROC curve of lasso regression analysis.

**Data S1:** Raw data of the study.

## Data Availability

The data that supports the findings of this study is available in the Data S1.
