# Supplementary material for: Factors Affecting Sleep Quality Among Pregnant Women: A Propensity Score‐Matched Analysis
Source: J Obstet Gynaecol Res. 2025 Dec 11;51(12):e70167. doi: 10.1111/jog.70167 (PMC12699176; doi:10.1111/jog.70167)
Supplement: Supplementary file 1 — Figure S1: Cross‐validation curve of lasso regression analysis. Figure S2: ROC curve of lasso regression analysis. [file JOG-51-0-s001.doc]

Supplementary figures


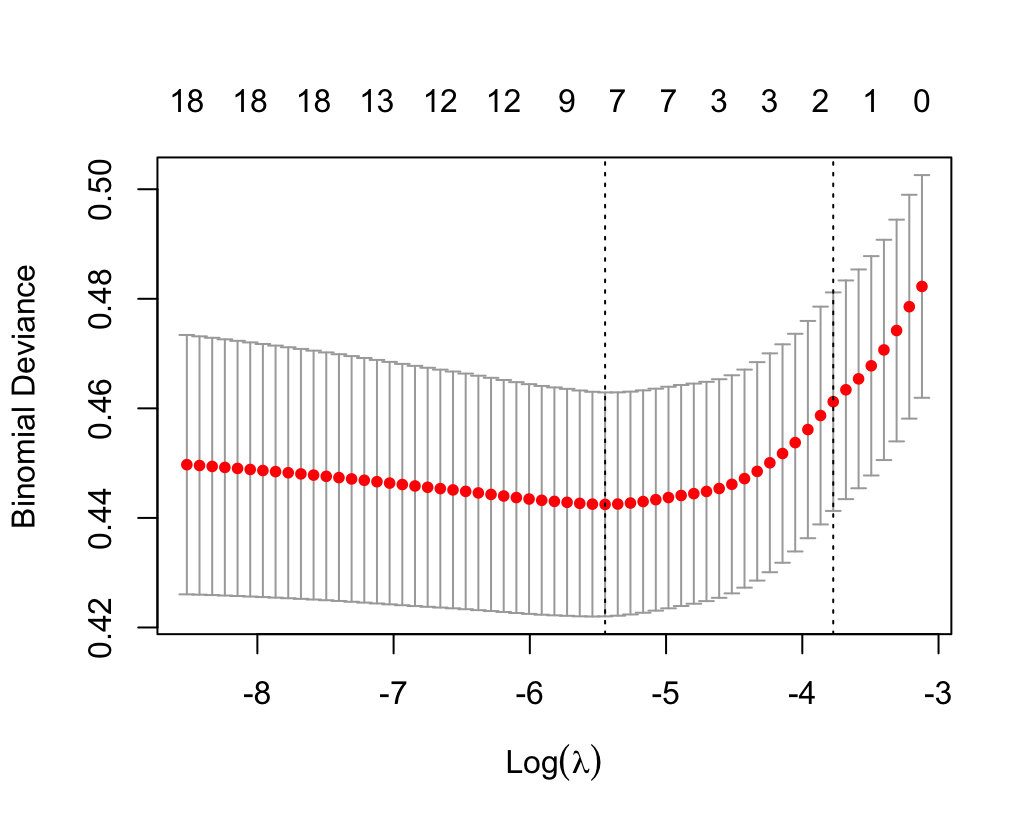


Figure S1 Cross-validation curve of lasso regression analysis


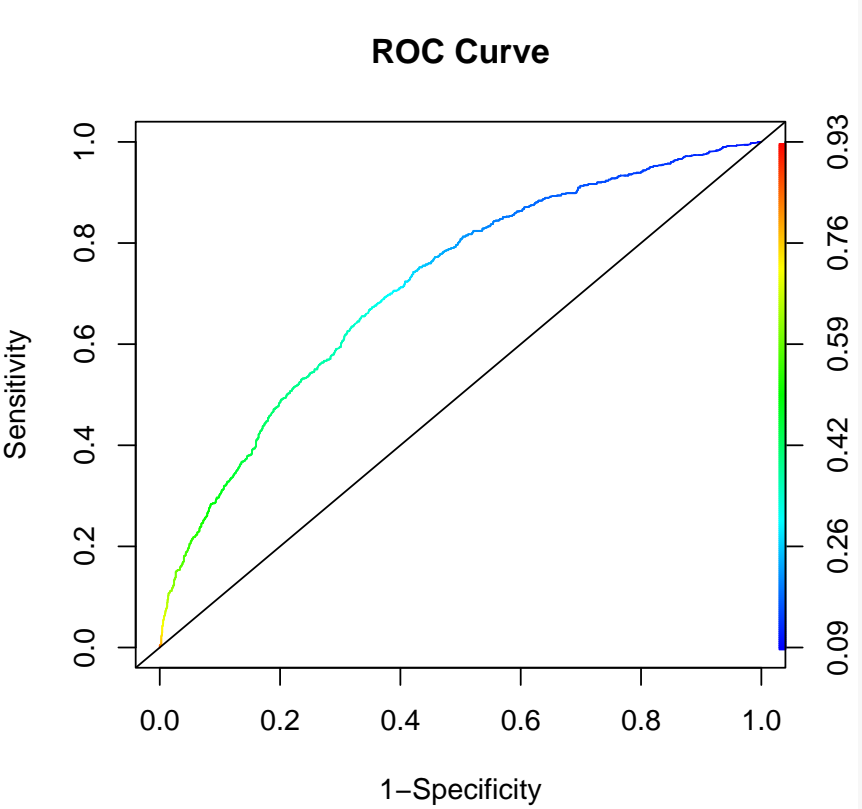


Figure S2 ROC curve of lasso regression analysis
